# Intestinal lymphangiectasia in a 3-month-old girl: A case report of Hennekam syndrome caused by CCBE1 mutation

**DOI:** 10.1097/MD.0000000000020995

**Published:** 2020-07-02

**Authors:** Antonella Fattorusso, Elena Sofia Pieri, Giovanni Battista Dell’Isola, Paolo Prontera, Elisabetta Mencaroni, Gabriela Stangoni, Susanna Esposito

**Affiliations:** a Pediatric Clinic, Department of Surgical and Biomedical Sciences, Università degli Studi di Perugia; b Center for Rare Diseases, Azienda Ospedaliera Santa Maria della Misericordia, Perugia; c Pediatric Clinic, Pietro Barilla Children's Hospital, Department of Medicine and Surgery, University of Parma, Parma, Italy.

**Keywords:** case report, CCBE1, CMV, FAT4, hennekam syndrome, intestinal lymphangiectasia

## Abstract

**Rational:**

Intestinal lymphangiectasia (IL) is a rare disease characterized by dilatation and rupture of intestinal lymphatic channels leading to protein-losing enteropathy. IL is classified as primary and secondary types.

**Patient concerns:**

A 3-month-old girl born at term from vaginal delivery with an APGAR score of 10/10 and birth weight of 4.310 g (>97° percentile) was admitted to our hospital because of increasing abdominal tenderness and diarrhea. At first examination, she presented an abdominal circumference of 60 cm, edema of the lower extremities and vulva, and facial dysmorphisms (hypertelorism, flat nasal bridge, flat mid-face).

**Diagnosis:**

Once admitted, ultrasonography showed a large amount of ascites, while blood laboratory investigations revealed severe hypoproteinemia, hypoalbuminemia and hypogammaglobulinemia. Lymphoscintigraphy with 99m-Tc-nanocolloid demonstrated abnormal leakage of the tracer in the abdomen as evidence of IL. To detect a possible secondary, exams were performed and demonstrated positive antibody titres for CMV-IgM and IgG in blood and CMV-DNA positivity in blood, urine, saliva, maternal milk, and gastric and duodenal biopsies. Genetic investigations identified the genomic variant c.472C>T of the CCBE1 gene, coding for a protein variant (p.Arg158Cys), in homozygosity.

**Interventions:**

Total parenteral nutrition was started and continued for a total of 18 days, then gradually bridged by enteral nutrition with a special formula. In addition, antiviral therapy for CMV infection was added first with intravenous ganciclovir for 14 days, resulting in the disappearance of blood viral load after 7 days of therapy and then with valganciclovir per os for another 30 days.

**Outcomes:**

The clinical course of the child gradually improved. A few days after starting treatments, lower extremities and vulvar edema disappeared, and abdominal circumference gradually decreased to a stable value of 38 cm, without any ultrasonographic signs of ascites left. Moreover, serum albumin and IgG rose to normal values after 3 months (4.3 g/dL and 501 mg/dL, respectively).

**Lessons:**

This case suggests that in presence of IL both primary and secondary causes should be evaluated. On the other hand, genetic diagnosis is crucial not only for diagnosis but also for prognosis in HS. Life expectancy and quality could deeply vary among different gene mutations and protein variants of the same gene. Further studies and case reports are needed to better understand the clinical meaning of these genetic results and the role of CMV as trigger of IL.

## 1 Introduction

Intestinal lymphangiectasia (IL) is a rare disease characterized by dilatation and rupture of intestinal lymphatic channels leading to protein-losing enteropathy .^[1,2]^ The major clinical features associated with this condition are severe hypoproteinemia, edema, chilous ascites, lymphocitopenia, hypogammaglobulinemia and diarrhea.^[3–5]^ Nutritional therapy is the most recommended treatment for IL, while octreotide has been proposed in association with diet.^[6]^ IL is classified as primary and secondary types: primary intestinal lymphangiectasia is caused by congenital abnormalities of lymphatic vessels and could be associated with genetic conditions such as Hennekam syndrome (HS); secondary intestinal lymphangiectasia (SIL) is caused by various circumstances that generally induce lymphatic obstruction or elevated lymph pressure, such as neoplastic, inflammatory or infectious diseases.^[3–5]^

HS is a rare autosomal recessive disorder caused by mutations in the CCBE1 or FAT4 genes.^[7]^ The incidence is approximately 1:100,000. Less than 50 cases have been reported in the literature, and the prevalence is unknown.^[8]^ The syndrome is characterized by multiple organ lymphangiectasia, dysmorphic facial appearance and mental retardation. Vascular and lymphatic vessel abnormalities resulting from genetic mutations lead to fluid accumulation, especially in the face, lower limbs and genitalia. The facial appearance is characterized by hypertelorism, large and depressed nasal bridges, round flat faces, puffy eyelids, tooth anomalies and small ears.^[9]^ Intellectual deficit is highly variable.^[8]^ Mutations in CCBE1 or FAT4 genes are responsible for the syndrome in 45% of cases (25% CCBE1 and 20% FAT-4).^[7,10]^ CCBE1 is involved in the migration of nascent lymphatic endothelial cells, providing migratory cues from the extracellular matrix, of which CCBE1 is thought to be a component. The function of FAT4 in lymphatic vasculature is still unknown.^[11]^

In this manuscript, we present a case of a 3-month patient with IL associated with a light form of HS, likely triggered by postnatal CMV infection.

## 2 Case presentation

### 2.1 Presenting concerns

A 3-month-old girl born at term from vaginal delivery with an APGAR score of 10/10 and birth weight of 4.310 g (>97° percentile) was admitted to our hospital because of increasing abdominal tenderness and diarrhea. This case report was approved by the Ethics Committee of the Umbria Region (PED-2019–01) and written informed consent was obtained from both parents. Parents also signed the consent for the publication of this case report.

### 2.2 Clinical findings

At first examination, she presented an abdominal circumference of 60 cm, edema of the lower extremities and vulva, and facial dysmorphisms (hypertelorism, flat nasal bridge, flat mid-face). She weighed 5.260 g and appeared neurologically adequate. No history of chronic diarrhea, poor growth or other gastrointestinal or neurological diseases was reported in her family (originally from Kosovo).

### 2.3 Diagnostic focus and assessment

**Figure 1:**
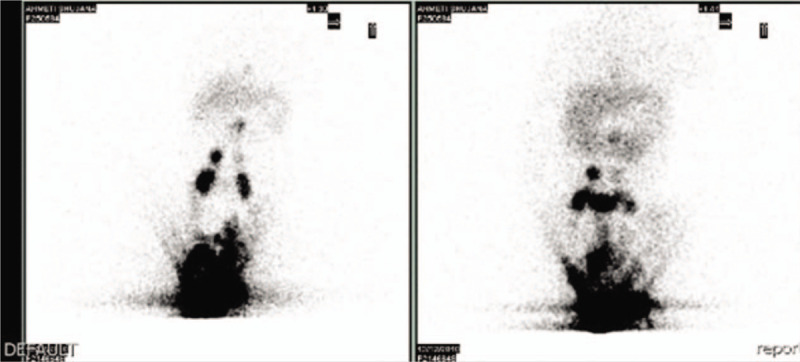
Lymphoscintigraphy with 99m-Tc-nanocolloid injected al level of the feet demonstrates abnormal leakage of the tracer in the abdomen. This result is suggestive of primary intestinal lymphangiectasia.

Once admitted, ultrasonography showed a large amount of ascites, while blood laboratory investigations revealed severe hypoproteinemia (3 g/dL, reference range 5.7–8 g/dL), hypoalbuminemia (1.7 g/dL, reference range 3.5–5.2 g/dL) and hypogammaglobulinemia with IgG <81 mg/dL (<2 standard deviation) and normal levels of IgM and IgA (52 mg/dL and 20 mg/dL, respectively). Other parameters, including complete blood count, C reactive protein, transaminase, blood urea nitrogen, creatinine, prothrombin time and activated partial thromboplastin time, were normal. Stool analysis showed the presence of a fair amount of lipids, while bacterial culture of stool specimens was negative. No evidence of liver dysfunction, renal loss or cardiac failure was found. A paracentesis was carried out, and the analysis of the aspirated fluid revealed chilous ascites (triglycerides, 528 mg/dL) with negative microbiological tests. Then, the toddler underwent lymphoscintigraphy with 99m-Tc-nanocolloid injected al level of the feet, which demonstrated abnormal leakage of the tracer in the abdomen as evidence of IL (Fig. 1).

To detect a possible infectious etiology of IL, additional investigations were performed. Tests for HIV, HCV, HBV, Epstein-Barr virus, herpes simplex and rubella virus were negative. Enzyme-linked immunoassay titre for CMV VCA-IgM and IgG were elevated (IgM 37.4 U/ml, n.v. <18; IgG 108 U/mL, n.v. <12). Real-time polymerase chain reaction (RT-PCR) showed CMV-DNA positivity in blood (3.9 × 10^3^ copy/mL), urine (40.2 × 10^3^ copy/mL), saliva (126.4 × 10^3^ copy/mL), and maternal milk (3.7 × 10^3^ copy/mL), recording a likelihood postnatal CMV infection via breast milk.

Gastroscopy and colonoscopy showed signs of edema and chronic inflammation with eosinophilic infiltrates in the duodenum and terminal ileum. CMV-DNA was detected by PCR on gastric and duodenal biopsies.

Due to the coexistence of lymphangiectasia and facial dysmorphisms, genetic tests were requested (CFTR, CCBE1 and FAT4 gene analysis). Sanger sequencing of amplified DNA on peripheral blood was performed, including exon and intron-exon junction analysis. Genomic variant c.472C>T of the CCBE1 gene, coding for a protein variant (p.Arg158Cys), was detected in homozygosity. No mutations in CFTR or FAT4 were detected.

### 2.4 Therapeutic focus and assessment

Total parenteral nutrition was started and continued for a total of 18 days, then gradually bridged by enteral nutrition with a special formula (Pregestimil 10% + Nidex 2% + MCT oil 1.5%), with low amounts of long chain fatty acids that was MCT-enriched. Octreotide treatment at a dosage of 5.7 mcg/kg/h by continuous intravenous infusion was carried out for 17 days. Short-term albumin infusion through a bolus of 1 mg/kg to raise serum albumin and protein levels was also administered.

In addition, antiviral therapy for CMV infection was added first with intravenous ganciclovir (60 mg/die, approximately 12 mg/kg/die) for 14 days, resulting in the disappearance of blood viral load after 7 days of therapy and then with valganciclovir per os (16 mg/kg b.i.d.) for another 30 days.

### 2.5 Follow-up and outcomes

The clinical course of the child gradually improved. A few days after starting treatments, lower extremities and vulvar edema disappeared, and abdominal circumference gradually decreased to a stable value of 38 cm, without any ultrasonographic signs of ascites left. Moreover, at discharge, serum albumin and IgG remained low (2.5 g/dL and 184 mg/dL, <2 standard deviation, respectively), but they rose to normal values after 3 months (4.3 g/dL and 501 mg/dL, respectively). Head magnetic resonance imaging, brainstem auditory evoked responses and fundus oculi examination were negative, ruling out CMV neurologic involvement.

## 3 Discussion

We have described a rare case of IL in a girl with a predisposing genetic condition (HS) and a simultaneous postnatal CMV infection. HS is an autosomal recessive disorder consisting of primary intestinal lymphangiectasia, facial anomalies, peripheral lymphedema, and mild to moderate levels of growth and intellectual disability.^[8,9]^ The role of CMV as a cause of secondary IL is still unknown, and only a few reports have noticed the association of CMV with IL,^[12–14]^ with only Nakase et al detecting CMV-DNA in the intestinal lesion.^[12]^ In none of previous reports, CMV infection was associated with HS. Our patient had a rapid clinical improvement after both nutritional treatment with a special formula for IL and antiviral therapy for CMV infection. It is likely that CMV infection triggered IL with ascites as the first and main feature of HS.

A very detailed clinical assessment led to the exclusion of the other secondary causes of IL. Endoscopic and bioptic investigations permitted to detect CMV-DNA on gastric and duodenal biopsies. MRI, BAER and fundus oculi exam did not show CMV neurologic involvement. However, the facial dysmorphisms noted in the girl (hypertelorism, flat nasal bridge and flat mid-face) associated with lymphangiectasia guided the research to a genetic cause. The genomic variant c.472C>T in the CCBE1 gene, which codes for a protein variant (p.Arg158Cys), was detected in homozygosity. This variant of CCBE1 seems to be related to a mild form of the syndrome and could explain either the rapid and stable remission of symptoms and the absence of neurological impairment.^[10]^

Strength of this case report is represented by the interesting association of IL with a primary cause as HS and a secondary trigger as CMV. Limitation is represented by the unclear role of CMV as trigger of IL in presence of a genetic disease as HS. However, from a clinical point of view we demonstrated the need to evaluate both primary and secondary etiologies in IL for appropriate management and prognosis information.

## 4 Conclusion

This case suggests that in presence of IL both primary and secondary causes should be evaluated in order to manage appropriately affected patients. In our patient, nutritional treatment for IL and antiviral therapy for CMV infection were successful. On the other hand, genetic diagnosis is crucial not only for diagnosis but also for prognosis in HS. Life expectancy and quality of life could deeply vary among different gene mutations and protein variants of the same gene. Further studies and case reports are needed to better understand the clinical meaning of these genetic results and the role of CMV as trigger of IL.

## Acknowledgment

We would like to thank all the pediatricians, geneticists and nurses involved in the management of this child, as well as the patient's parents.

## Author contributions

AF and ESP wrote the first draft of the manuscript; GBDI performed the literature review; PP and GS performed the genetic diagnosis; EM was in charge of the patient's follow-up; SE supervised the patient's management, critically revised the paper and gave her scientific contribution. All the authors read and approved the final version of the manuscript.
